# Intentions of Environmentally Friendly Behavior Among Sports Club Members: An Empirical Test of the Theory of Planned Behavior Across Genders and Sports

**DOI:** 10.3389/fspor.2021.657183

**Published:** 2021-05-31

**Authors:** Michael Braksiek, Tim F. Thormann, Pamela Wicker

**Affiliations:** ^1^Department of Sports Science, University of Vechta, Vechta, Germany; ^2^Department of Sports Science, Bielefeld University, Bielefeld, Germany

**Keywords:** behavioral intentions, environmental attitudes, sustainability, voluntary sports club, grassroots sports, climate change

## Abstract

Environmentally friendly behavior has become increasingly important in recent years to reduce the speed of climate change and its negative impacts. Individual behavior, including environmentally friendly behavior, is largely formed by behavioral intentions. This study draws on the theory of planned behavior to examine the effects of attitudes toward the behavior, subjective norms, and perceived behavioral control on intentions of environmentally friendly behavior. It also investigates differences between genders and among sports. The study is based on data from a nationwide online survey of community sports club members in Germany in five team/racket sports (*n* = 3,036). Existing measures to operationalize the constructs were adapted to the present research context. The data were analyzed using structural equation modeling. The results show that the theoretical assumptions of the theory of planned behavior were largely supported by the data, implying that the antecedents of environmentally friendly behavioral intentions can be applied to club members. Furthermore, gender- and sports-specific differences in the antecedents–intention relationship were detected. This study is among the first to examine environmentally friendly behavioral intentions in community sports clubs. It adds to an increasing body of research investigating environmental sustainability in sports.

## Introduction

The United Nations (UN) have developed 17 sustainable development goals which are indicative of the most pressing issues worldwide (UN, 2020b). Goal 13 suggests to “take urgent action to combat climate change and its impacts” (UN, 2020a, n.p.), recognizing that “2019 was the second warmest year on record and the end of the warmest decade (2010–2019) ever recorded” (UN, 2020a, n.p.). Climate change is globally relevant as not only does it affect all countries in terms of economic aspects, but it also influences people's lives as weather events tend to become more extreme (UN, 2020a). The Intergovernmental Panel on Climate Change (IPCC) has outlined that climate change poses risks to humans and natural systems (IPCC, 2018), indicating the need for combating the drivers behind climate change. Climate change is caused by global warming which, in turn, is caused to a large extent by human activities, such as traveling and industrial production, that result in emissions. Hence, the IPCC (2018) strongly recommends undertaking efforts to avoid global warming of 1.5°C or higher compared to preindustrial levels and to engage in environmentally sustainable behaviors that reduce emissions. It has identified a number of synergies between Goal 13 and other sustainable development goals, such as responsible consumption and production as well as good health and well-being (IPCC, 2018).

Sports participation is one of the activities that contribute to individuals' health and well-being (e.g., Humphreys et al., 2014; Downward et al., 2016; Orlowski and Wicker, 2018; Wicker, 2020). However, practicing sports produces not only positive externalities like health and well-being but also negative externalities such as environmental impacts by deploying natural resources and producing emissions (e.g., Wicker, 2019; McCullough et al., 2020a,b). Accordingly, the review by Trendafilova and McCullough (2018) indicates that a number of studies have been conducted examining the environmental impacts of sports and environmentally sustainable behavior in sports. However, Carmichael (2020) stresses that the focus of previous research was on professional sports organizations and elite sports events, hence largely neglecting sports at the grassroots level.

The present study's research context is grassroots-level and voluntary sports clubs. Sports clubs represent the most important provider of organized sports in many European countries and are home to millions of members and participants (Breuer et al., 2015). Following the annual statistics of the German Olympic Sports Confederation (DOSB), about 88,000 sports clubs encompass 27.8 million memberships alone in Germany (DOSB, 2020), with most members actively practicing sports in their clubs. Thus, grassroots sports clubs represent an organizational context where pro-environmental behavior is relevant (McCullough et al., 2020b). However, organizations such as sports clubs also depend on their members' willingness to behave in environmentally friendly ways. Hence, it is critical to understand members' intentions of environmentally friendly behavior and what factors form these intentions.

The purpose of this study is to examine the antecedents of environmentally friendly behavioral intentions among community sports club members through the lens of the theory of planned behavior (TPB). This theory has been widely used to examine behavioral intentions in a number of contexts and is applied to the research context of grassroots sports clubs in this study. The empirical analysis is based on a cross-sectional survey of sports club members in five team/racket sports in Germany (i.e., football, basketball, handball, ice hockey, and tennis) which are typically characterized by possessing club houses and formal training facilities where all members have the opportunity to perform environmentally sustainable behavior or not (e.g., switching off lights, reducing water consumption in the shower, separating waste). This study advances the following two research questions: (1) to what extent can the antecedents of environmentally friendly behavioral intentions as suggested by the TPB be applied to sports club members? And (2) are there differences in the antecedents–intention relationship between both genders and among sports? Such an examination enhances our understanding of the drivers behind behavioral intentions in relation to environmentally sustainable behavior and the potential of grassroots sports clubs to contribute to the environmental sustainability of the sports sector. The study contributes to the increasing body of research on sports ecology.

## Theoretical Framework and Literature Review

### Sports Environmental Research

Sports environmental research emerged early in the 1980s, but until 2008, only a few occasional and qualitative studies examining the role of the natural environment in the sports context were published (for a review see Mallen et al., 2011). From 2008 onward, the growing public interest in environmental problems was accompanied by proportionally growing research in the field of sports environmental research (Dingle, 2017). While most studies in the early stage tried to answer questions about why and how environmental strategies are applied within sports (e.g., Trendafilova et al., 2013), studies of pro-environmental behavior of spectators and sports participants have attracted the interest of researchers (e.g., McCullough and Cunningham, 2011; Wicker, 2019). Most recently, a group of scholars has grouped the different environmental approaches in the existing literature under the subdiscipline of sports ecology (McCullough et al., 2020a). Previous research in the field of sports ecology was two-fold. The first and most often investigated perspective is the impact of sports on the environment (e.g., Wicker, 2018, 2019; McCullough et al., 2020b), while the second approach focused on the impact of environmental changes on sports (Orr and Inoue, 2019; Orr, 2020).

Investigating pro-environmental intentions and behavior in the sports context falls within the former perspective. Previous studies investigated the pro-environmental intentions and environmentally friendly behavior of sports spectators (e.g., McCullough and Cunningham, 2011; Inoue and Kent, 2012), athletic departments (Casper et al., 2014), sports students (Casper and Pfahl, 2012), sports tourists (Wicker, 2018), and active sports participants (Trail and McCullough, 2018; Wicker, 2019). In previous research, environmental values, beliefs, and personal norms positively determined pro-environmental intentions among college sports spectators not only at the sports event but also in their everyday life (Casper et al., 2014). More recently, personal values were shown to positively impacted personal and sports norms. However, only sports norms had a positive influence on the recycling intentions of collegiate basketball spectators in the form of higher perceived recycling benefits, higher perceived environmental efforts of the athletic department, and lower perception of recycling inconvenience (Casper et al., 2020). Moreover, environmental values positively influenced beliefs which in turn affect personal norms among undergraduate students. The students' personal norms finally influenced their pro-environmental behavior in the form of conservation in the personal and organizational sphere (Casper and Pfahl, 2012), suggesting that environmental attitudes and subjective norms also affect actual behavior.

### The Theory of Planned Behavior

The TPB, proposed by Ajzen (1991), has its origin in the field of social psychology. It emerged from the theory of reasoned action (Fishbein and Ajzen, 1977) and extended the latter to more specifically approach the origin of human behavior. In order to understand the applicability of the TPB for explaining pro-environmental behavior, it is crucial to first elucidate on the theory of reasoned action.

According to the theory of reasoned action, behavioral intentions are the immediate antecedent to behavior which is mostly under volitional control. The concept of behavioral intentions is defined as “motivational factors that influence a behavior; they are indications of how hard people are willing to try, of how much of an effort they are planning to exert, in order to perform the behavior” (Ajzen, 1991, p. 181). In the context of this study, intention is understood as the willingness of sports club members to perform pro-environmental behavior. Empirical results from multiple experiments confirmed the positive relationship between behavioral intentions and actual behavior (Webb and Sheeran, 2006). Behavioral intentions, in turn, are a function of salient beliefs which are divided into behavioral and normative beliefs (Madden et al., 1992). Behavioral beliefs can be seen as an underlying influence of an individual's attitude toward a behavior, whereas normative beliefs affect subjective norms. Consequently, behavioral intentions are determined by an individual's attitudes and subjective norms that serve as antecedents of intentions.

Attitudes can be seen as the expression of beliefs and values toward a specific area (Fishbein and Ajzen, 1977). They are linked to positive or negative outcomes or experiences of individuals with a certain behavior and describe the internal component of intentions (Fishbein and Ajzen, 1977). Applied to the present research context, positive experiences with regard to saving energy or recycling waste could lead to positive attitudes toward pro-environmental behavior and, therefore, increase the intention to behave in an environmentally friendly manner.

The external component of intentions is described as subjective norms which are influenced by the social pressure of friends, family, community members, or society as a whole (Fishbein and Ajzen, 1977; Kaiser et al., 2005). Subjective norms express the thoughts of individuals who are expected by their social networks to showcase specific behavior. Usually, individuals try to meet the expectations of important social ties. For example, if parents are strictly against smoking, the intention to smoke is rather low (Ajzen, 1991). Overall, the more positive an attitude individuals have toward a certain behavior and if they think others would be appreciative when they perform this particular behavior (subjective norms), the higher is the intention and the more likely is the individual to perform the respective behavior.

The strength of the relationship between intentions and behavior is limited by three conditions. First, the congruence between the specificity of the intention and the measurement of behavior influences the magnitude of the relationship. Second, the temporal stability of the intentions between measurement and actual behavior is important. Finally, the magnitude of volitional control the individual has to perform the behavior is relevant. The latter represents a decisive limitation of the theory of reasoned action, because it ignores the magnitude of volitional control which, however, reflects another important antecedent of intentions and behavior (Ajzen, 1991).

Tackling this limitation, the TPB extends the theory of reasoned action by the construct of perceived behavioral control which serves as a direct predictor of intentions, but also behavior (Ajzen, 1991). Perceived behavioral control reflects individuals' perceptions of their ability to actually perform a behavior. It consists of requisite resources and opportunities that are available to a person in a specific situation (e.g., time, money, opportunity to perform behavior, Whitehead and Wicker, 2018, 2019). Thus, perceived behavioral control also reflects the perceived difficulty to perform a specific behavior (Ajzen, 1991). The inclusion of perceived behavioral control also impacts the effect of attitudes and subjective norms on behavioral intentions. Even though attitudes and subjective norms toward pro-environmental behavior may be positive, the intention to behave environmentally friendly can be harmed by time or monetary restrictions (e.g., using public transport or riding a bike to the club is too time-consuming), which makes it impossible for the individual to perform the respective behavior.

### Application of the Theory of Planned Behavior to Sports Environmental Research

The TPB has been largely applied to environmental research (e.g., Clement et al., 2014) and sports research (e.g., Whitehead and Wicker, 2018, 2019) separately, but has rarely been applied to sports environmental research (e.g., McCullough and Cunningham, 2011; McCullough, 2013).

Within environmental research, attitudes, subjective norms, and perceived behavioral control positively predicted the intentions for different types of energy conservation (Clement et al., 2014), environmental behavioral intentions in the workplace (Greaves et al., 2013), intentions to visit green hotels (Han et al., 2010), and the likelihood to purchase environmentally friendly products (Maichum et al., 2016). Furthermore, the positive relationship between intentions and actual behavior was confirmed for energy conservation (Macovei, 2015) and everyday pro-environmental behavior such as switching off lights (Levine and Strube, 2012). In sports research, the three constructs predicted the intention to attend sports events (e.g., Cunningham and Kwon, 2003), the intention to use doping substances (e.g., Ntoumanis et al., 2014), the intention to participate in recreational sports activities (e.g., Alexandris and Stodolska, 2004; Chuan et al., 2014), and the intention to recycle products (McCullough and Cunningham, 2011).

The TPB has been largely neglected in recent years to explain pro-environmental intentions or behavior in the sports context with two exceptions (McCullough and Cunningham, 2011; McCullough, 2013). Both studies used the theory to investigate recycling intentions of sports spectators in college sports (McCullough, 2013) and in youth baseball (McCullough and Cunningham, 2011) with slightly different results. McCullough (2013) showed that college sports spectators' attitudes, subjective norms, and perceived behavioral control influenced their intention to recycle. The investigation of youth baseball spectators showed that previous behavior and subjective norms positively influenced their recycling intention. However, attitudes and perceived behavioral control had no significant association with recycling intentions (McCullough and Cunningham, 2011). This finding was explained by the scarce accessibility of recycling containers at the event location which was also the main obstacle to recycle for college sports spectators (McCullough, 2013). Even though Trail and McCullough (2018) did not explicitly rely on the TPB, they also showed that internal and external constraints such as the lack of interest of others, which is comparable to subjective norms in the TPB, influenced the sustainability intentions of running event participants.

Collectively, this overview suggests that the TPB has only been rarely applied to sports environmental research and mainly concentrated on recycling intentions. This study tests the applicability of the TPB beyond sports spectators' recycling intentions. It tries to enhance our understanding of the influence of attitudes, subjective norms, and perceived behavioral control on pro-environmental intentions of sports club members. Based on the TPB, positive effects of attitudes, subjective norms, and perceived behavioral control on pro-environmental intentions of sports club members are expected. Furthermore, gender differences and differences between sports with regard to the influence of attitudes, subjective norms, and perceived behavioral control are investigated.

### The Role of Gender

The role of gender has been widely discussed in environmental research, with several theoretical explanations being advanced as to why women tend to act more environmentally friendly than men. According to the concept of ecofeminism, gender differences in environmental behavior occur due to different conceptualizations of the world (Sakellari and Skanavis, 2013). The psycho-cultural approach explains the closeness of women and nature through a common history of oppression by patriarchal institutions. Patriarchy characterizes men as dominant and controlling, while the female identity is described as emotional and caring. Additionally, environmental problems seem to affect women earlier and more directly than men (Estévez-Saá and Lorenzo-Modia, 2018). The close connection between women and nature results in stronger caring for environmental problems (Leach, 2007). Furthermore, higher awareness of environmental problems has led to stronger engagement of women in environmental politics, which influences the experiences of girls and women (Sakellari and Skanavis, 2013).

The social approach emerged from the social role theory and explains gender differences in environmental behavior through different social roles for women and men (Eagly and Wood, 1991; Wood and Eagly, 2012). Generally, these roles emerge from psychological and social processes starting from the division of labor, which is, in turn, affected and reproduced by various economic, cultural, and biological factors (Wood and Eagly, 2012). Accordingly, women are assigned to family roles, including activities such as child-rearing and domestic work that again lead to a stronger caring ethic, not only for other human beings but also for the natural environment (Eagly and Wood, 1991; Wood and Eagly, 2012). Several empirical studies confirmed these theoretical assumptions and showed stronger environmentally friendly intentions of women in different research contexts, including college sports (Israel and Levinson, 2004; Casper et al., 2017).

## Methods

### Data Collection

Data were collected via a nationwide online survey among non-profit sports club members from five different sports in Germany, namely, football, basketball, handball, ice hockey, and tennis. The online survey was programmed on the platform SoSci Survey which can be used free of charge for scientific purposes. Various distribution channels were used to reach as many sports club members as possible, including distribution through social media like Facebook and Instagram, newsletters to club officials, and online newspapers. In the absence of a registry of sports club members from which a random sample could be drawn, convenience sampling was employed—similar to previous sports environmental research (e.g., Casper and Pfahl, 2012).

The online survey was finished by 3,329 sports club members across sports. However, it was not possible to calculate a response rate because an undefined number of clubs were approached. During the data cleaning process, 293 observations had to be excluded from the sample. The reasons for exclusion covered the same answers to multiple consecutive questions, speeding, and failed plausibility as well as internal validity checks. The final sample for the empirical analysis is *n* = 3,036 sports club members.

### Sample Characteristics

Table 1 gives an overview of the sample characteristics by sports and gender. Altogether, 67% of respondents are male. In the full sample, average age was 32.16 years (SD = 14.61). Female respondents (M_age_ = 29.33; SD = 13.07) are slightly younger than their male counterparts (M_age_ = 33.57; SD = 15.11).

**Table 1:** Number of respondents by sport and gender.

**Sport**	**Gender**		**Total**
	**Women**	**Men**	
Tennis	213	473	686
Ice hockey	71	393	464
Basketball	220	413	633
Handball	188	188	376
Football	297	580	877
Total	989	2,047	3,036

### Measures and Variables

Table 2 displays the variables and their descriptions. To measure the TPB components, three to five items for each construct were developed based on Ajzen (2002) suggestions for developing direct measures of the TPB constructs. Items for direct measures have a clear focus on the target behavior. Unlike indirect (belief-based) measures, they do not require qualitative pilot work for eliciting respondents' beliefs (Ajzen, 2002, 2019). We adapted the concrete items suggested by Ajzen (2002) to the target behavior of acting environmentally friendly within the context of community sports clubs. The items were worded in a way that they were applicable across various team/racket sports and sports clubs to ensure that the survey is considered relevant across the country. On the contrary, adapting the items to specific environmentally friendly actions of particular sports clubs would have limited the reach of the survey, the number of potential respondents, and the generalizability of the findings, respectively.

**Table 2:** Overview of variables.

**Variable**	**Description**	**Mean**	**SD**
**Attitudes toward the behavior: Generally I believe that environmentally-friendly behavior in my SPORT club is …**			
att1	(1 = harmful; 5 = beneficial)	4.16	0.864
att2	(1 = unpleasant; 5 = pleasant)	3.76	1.138
att3	(1 = bad; 5 = good)	3.89	1.200
att4	(1 = worthless; 5 = valuable)	4.15	0.886
att5	(1 = unenjoyable; 5 = enjoyable)	3.56	1.094
**Subjective norms (1** **=** **totally disagree; 5** **=** **totally agree)**			
sn1	Most people who are important to me think believe I should act environmentally friendly	3.50	0.983
sn2	Presumably my fellow club members expect me to act environmentally friendly	3.05	1.099
sn3	In my SPORT club, it is appreciated when I act environmentally friendly	3.16	1.077
sn4	In my SPORT club, people often act environmentally friendly	3.06	0.922
**Perceived behavioral control (1** **=** **totally disagree; 5** **=** **totally agree)**			
pbc1	I am convinced that I can act environmentally friendly in my SPORT club when I want to	3.82	0.942
pbc2	It is in my hands whether or not I act environmentally friendly in my club	3.80	1.068
pbc3	Environmentally friendly behavior is easily possible in my club	3.44	1.030
**Behavioral intentions (1** **=** **totally disagree; 5** **=** **totally agree)**			
int1	I would like to take every opportunity to act environmentally friendly in the next 2 weeks	3.70	0.959
int2	It is my goal to take every opportunity to act environmentally friendly in the next 2 weeks	3.46	0.919
int3	I will definitely try to take every opportunity to act environmentally friendly in the next 2 weeks	3.55	1.060

*SPORT was replaced by the corresponding type of sports in the sports-specific surveys*.

In this study, environmentally friendly behavior was understood as “behavior that consciously seeks to minimize the negative impact of one's actions on the natural and built world” (Kollmuss and Agyeman, 2002, p. 240). The literature distinguishes different dimensions of environmentally friendly behavior, including recycling, sustainable consumption, energy saving, and transportation (Preisendörfer, 1999). The survey included these aspects, and respondents were provided with an explanation of environmentally friendly behavior. Specifically, the introduction to the questions for the TPB part of the survey included the following description: “In this context environmentally-friendly behavior are actions such as waste separation, ecological consumption (e.g., using reusable bottles), saving energy (e.g., energy and water saving), as well as the use of environmentally friendly transportation (e.g., carpooling).” Hence, respondents were informed about what was considered environmentally friendly behavior in the survey.

Turning to the concrete measures in the survey, attitudes toward environmentally friendly behavior in the sports club were assessed using five five-point semantic differential items (Ajzen, 2002). Semantic differentials are a common method to measure attitudes (Fishbein and Ajzen, 2010). Respondents rate an attitude object (here, environmentally friendly behavior) on a set of bipolar evaluative adjective scales. According to Fishbein and Ajzen (2010), semantic differentials generally include instrumental (e.g., *bad–good*) and experiential (e.g., *unpleasant–pleasant*) items but can measure attitude as a unidimensional construct. Therefore, the items of a semantic differential are meant to correlate and load on a single factor in a confirmatory factor analysis (CFA) for valid and consistent measurement of attitude as a latent construct. Items that do not load on the latent attitude factor should be removed as they do not represent the latent construct (Fishbein and Ajzen, 2010). The initial scale of the survey consisted of three instrumental and two experiential items. A CFA for assessing the suitability of the scale as a measurement model for the subsequent structural equation model (SEM) yielded poor model fit. Two items (att1 and att4) correlated moderately with each other (r = 0.63; *p* < 0.001), but low with the other items (r = 0.30 to 0.36; *p* < 0.001). The corrected item-total correlations were moderate (0.48 and 0.50) (Hinkle et al., 2003). Accordingly, the factor loadings of the two items (att1 and att4) were small and not significant (0.045 and 0.052). Therefore, the two items were removed. The resulting scale consisted of two experiential and one instrumental item, hence both attitudinal dimensions were covered and, therefore, content validity could be assumed (Fishbein and Ajzen, 2010). The two experiential items (att2 and att5) were moderately correlated (r = 0.68, *p* < 0.001). The instrumental item (att3) was highly correlated with one (att2; r = 0.76, *p* < 0.001) and moderately correlated with the other experiential item (att5; r = 0.63, *p* < 0.001). As the experiential items were only moderately correlated and the instrumental item measured an additional part of the attitude, the items represent attitudes as a latent construct without being redundant. Since there were only three indicators left, no global or incremental model fit could be investigated, but the local model fit (significant and high factor loadings; Heene et al., 2012) and the internal consistency (α = 0.871) of the scale were good (Nunnally and Bernstein, 1994).

Subjective norms related to environmentally friendly behavior in the sports clubs were measured with four items using five-point Likert scales. The first three items measured the injunctive and the last item the descriptive aspects of subjective norms (Fishbein and Ajzen, 2010). A CFA for assessing the suitability of the scale as measurement model for the SEM yielded poor model fit. According to the factor loadings, one item for the injunctive aspect (sn1) was removed. In contrast to the other items, this item did not refer directly to the sports club as social context. The internal consistency of the scale was acceptable (α = 0.756), and the local model fit was good (Nunnally and Bernstein, 1994).

PBC was measured with three items using five-point Likert scales. Each item consisted of a positive statement about having control over acting environmentally friendly in the sports club. The internal consistency of the scale was acceptable (α = 0.753), and the local model fit was good (Nunnally and Bernstein, 1994).

Behavioral intentions to act environmentally friendly were captured with three items using five-point Likert scales. Each item consisted of a positive statement about the intention to act environmentally friendly in the sports club in the next 2 weeks. The internal consistency (α = 0.856) and the local model fit of the scale were good (Nunnally and Bernstein, 1994).

### Statistical Analysis

The data were prepared, and descriptive statistics were calculated in SPSS 26 (IBM Corp., 2019). The statistical analyses were conducted using Mplus 7.4 (Muthén and Muthén, 2017). The latent analyses were based on a maximum likelihood (ML) estimator with standard errors and a mean- and variance-adjusted chi-square test statistic that are robust to non-normality (MLMV; Muthén and Muthén, 2017). Currently, the MLMV seems to be the most effective ML estimator (Maydeu-Olivares, 2017). The data set was complete; hence, no missing values had to be dealt with.

In a first step of the analysis, the measurement invariance of the TPB constructs was investigated in order to determine if the scales measure the same constructs in all subsamples. Otherwise, meaningful comparisons of means and regression coefficients across different groups cannot be made (Chen, 2008). Multigroup confirmatory factor analyses (MG-CFA) were employed to test for measurement invariance. Successively, configural invariance (equal factor structure), metric invariance (equal factor loadings), and scalar invariance (equal intercepts) were examined. At least, scalar invariance is needed to compare means and coefficients. The criterion of change in fit indices between the different model restrictions was used: ΔCFI ≥ 0.01 supplemented by ΔRMSEA ≥ 0.015 or ΔSRMR ≥ 0.030 indicates non-invariance of the metric model, and ΔCFI ≥ 0.01 supplemented by ΔRMSEA ≥ 0.015 or ΔSRMR ≥ 0.010 indicates non-invariance of the scalar model (Chen, 2007). The MG-CFAs were also used to compare the latent factor means of the TPB constructs across the subsamples, if (partial) scalar invariance held. In comparison to latent means with MG-CFAs, one group is set as reference group with a latent factor mean constrained to 0. The other groups are tested against the reference group at a 5% significance level. Hedge's g was calculated as effect size to respect the different sample sizes (Hedges, 1981).

In a second step, a SEM was estimated in each subsample. The hypothesized paths of the SEM were in accordance with the predictions of the TPB. The SEMs were evaluated according to common conventions: RMSEA ≤ 0.05 or ≤ 0.08 and SRMR ≤ 0.05 or ≤ 0.010 for a good or acceptable model fit, CFI and TLI ≥ 0.95 or ≥ 0.90 for a good or acceptable model fit, a lower limit of RMSEA confidence interval close to 0 and an upper limit of ≤ 0.08 for a good model fit, and a non-significant test for the closeness of fit (Schermelleh-Engel et al., 2003; Hooper et al., 2008).

## Results

### Measurement Invariance (Multi-Group SEM/CFA)

Given the adequacy of the measurement models in the full sample, multigroup CFAs were conducted to determine whether the measurement models for the TPB variables were invariant across sports and gender. The measurement models for attitude, subjective norms, and PBC were tested simultaneously, and the inter-factor correlations were estimated freely, since they were also used in the later SEM in that way. Table 3 shows the corresponding results.

**Table 3:** Goodness-of-fit statistics and model comparisons for the multigroup confirmatory factor analyses.

**Model**	**CFI**	**Δ CFI**	**RMSEA**	**Δ RMSEA**	**SRMR**	**Δ SRMR**
**Gender**						
Configurial	0.972		0.058		0.034	
Metric	0.971	−0.001	0.055	−0.003	0.036	−0.002
Scalar	0.968	−0.003	0.055	0.000	0.038	−0.002
**Sport**						
Configurial	0.968		0.056		0.040	
Metric	0.962	−0.006	0.056	0.000	0.046	−0.006
Scalar	0.952	−0.010	0.058	−0.002	0.051	−0.005

*CFI, comparative fit index; RMSEA, root mean square error of approximation; SRMS, standardized root mean square residual*.

The measurement models yielded good model fits for both subsamples under all conditions for invariance. Relating to the invariance across gender, the deterioration of the fit indices was small and under the cutoff values for non-invariance. Concerning the invariance across sports, the decrease of the CFI from the metric to the scalar model was very slightly below the cutoff value. Since the other limits were clearly not exceeded, invariance can be assumed here as well. In summary, the measurement models for attitude, subjective norms, and PBC were scalar invariant across genders and sports.

The invariance of the measurement model for behavioral intentions was examined separately. Since the measurement model had only three indicators and therefore no degrees of freedom in the configural model, the model fit for this model was perfect. Therefore, the criterion of change in fit indices could not be applied here and only the fit indices of the scalar models could be investigated. For gender, the very good model fit of the scalar model (CFI = 1.000; RMSEA = 0.002; SRMR = 0.011) indicated invariance across genders. Regarding sports, the poor model fit of the scalar model (CFI = 0.770; RMSEA = 0.326; SRMR = 0.183) indicated non-invariance across sports.

### Latent Mean Comparisons

For the comparisons of the latent means between women and men as well as between the different sports, the MG-CFAs for evaluating the measurement invariance were used. More precisely, the scalar model provided latent means and additional statistics for a comparison with a reference group.

The latent mean differences between genders and sports are shown in Table 4. In the gender-specific comparison of latent means, women were set as the reference group. The men had a significantly more positive attitudes toward environmentally friendly behavior in sports clubs (*M* = 0.084; *p* = 0.048; *g*_*Hedges*_ = 0.054) as well as a significantly higher PBC over acting environmentally friendly in sports clubs (*M* = 0.120; *p* = 0.005; *g*_*Hedges*_ = 0.075). The subjective norms related to acting environmentally friendly in sports clubs were perceived significantly weaker by men (*M* = −0.126; *p* = 0.003; *g*_*Hedges*_ = 0.079). Men also had significantly weaker intentions to act environmentally friendly in sports clubs in the next 2 weeks (*M* = −0.263; *p* < 0.001; *g*_*Hedges*_ = 0.173).

**Table 4:** Latent mean differences (factor means) between sports.

	**Attitude**	**Subjective norm**	**PBC**	**Intention**
**Gender**				
Female	0.000	0.000	0.000	0.000
Male	0.084^*^	−0.126^**^	0.120^**^	−0.263^***^
**Sport**				
Tennis	0.000^b^^c^^d^^e^	0.000^b^^c^^d^^e^	0.000^b^^c^^d^^e^	0.000^b^^c^^d^^e^
Ice hockey	−0.531^a^^c^^d^^e^	−0.695^a^^c^	−0.420^a^	−0.306^a^^c^
Basketball	−0.102^a^^b^^d^^e^	−0.445^a^^b^^d^^e^	−0.349^a^	−0.116^a^^b^^d^^e^
Handball	−0.392^a^^b^^c^^e^	−0.709^a^^c^	−0.424^a^	−0.428^a^^c^
Football	−1.243^a^^b^^c^^d^	−0.654^a^^c^	−0.347^a^	−0.373^a^^c^

* *p < 0.05*;

** *p < 0.01*;

*** *;p < 0.001*;

a *significantly (p < 0.05) different from tennis*;

b *significantly different from ice hockey*;

c *significantly different from basketball*;

d *significantly different from handball*;

e *significantly different from football*.

In the latent mean differences between sports, the MG-CFAs for the evaluation of the measurement invariance could only be used for the comparisons with the tennis sample, as this group was the reference group in these analyses. For the other comparisons, further MG-CFAs with other reference groups were conducted. As scalar invariance did not hold for the intentions scale, the means of intentions were compared using the alignment method (Asparouhov and Muthén, 2014). This method allowed valid comparison of the latent factor means even if there was no scalar invariance, and it could identify non-invariant parameters as well (Marsh et al., 2018).

All differences in attitudes between sports were statistically significant. Tennis club members had the most positive and football club members the most negative attitudes toward acting environmentally friendly in their sports club. Tennis club members also perceived the significantly strongest norms to act environmentally friendly in their club compared to all other sports. Additionally, basketball club members perceived significantly stronger norms than ice hockey, handball, and football club members. Ice hockey, handball, and football club members did not differ regarding subjective norms. Tennis club members had the significantly strongest PBC over acting environmentally friendly in their club compared to all other sports. The other sports did not differ among each other. For behavioral intentions, the results were the same as for subjective norms: tennis club members had the significantly strongest intentions to act environmentally friendly in their sports club compared to all other sports. Basketball club members had significantly stronger intentions than ice hockey, handball, and football club members. Ice hockey, handball, and football club members did not differ regarding their intentions. Hedge's g was small (<0.2) for all differences.

### Structural Equation Models

**Figure 1:**
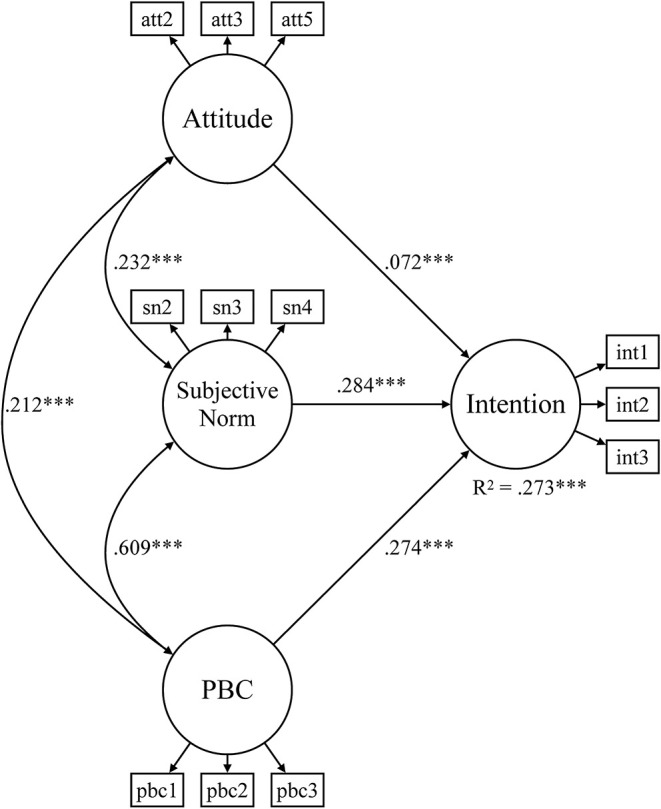
Structural equation model for environmentally friendly behavioral intentions (full sample). PBC, perceived behavioral control; ***p < 0.001.

According to the predictions of the TPB, a structural equation model was estimated for the full sample and the gender- and sport-specific subsamples. The intention to act environmentally friendly in the sports club was set to be predicted by attitudes, subjective norms, and PBC, which were free to correlate. Table 5 summarizes the goodness-of-fit indices for each model. The fit indices indicated a good model fit in the full sample and in each subsample. Figure 1 shows the SEM for the full sample. All relationships are positive and statistically significant. Attitudes, subjective norms, and PBC explain 27.3% of the variation in behavioral intentions. Thus, the theoretical assumptions of the TPB were supported by the data.

**Table 5:** Fit indexes of the estimated structural equation models.

**Model**	**CFI**	**TLI**	**RMSEA**	**RMSEA 90% CI**	**pclose**	**SRMR**	**χ^2^**	**df**	***p***
Full sample	0.971	0.960	0.050	0.045; 0.054	0.527	0.035	408.661	48	< 0.001
Women	0.962	0.948	0.056	0.048; 0.065	0.094	0.040	198.784	48	< 0.001
Men	0.975	0.966	0.044	0.039; 0.050	0.948	0.034	241.954	48	< 0.001
Tennis	0.966	0.953	0.046	0.036; 0.057	0.723	0.040	117.560	48	< 0.001
Ice hockey	0.975	0.965	0.045	0.031; 0.059	0.710	0.041	93.199	48	< 0.001
Basketball	0.976	0.967	0.037	0.025; 0.049	0.966	0.042	89.741	48	< 0.001
Handball	0.971	0.960	0.042	0.025; 0.058	0.789	0.047	79.481	48	0.003
Football	0.981	0.974	0.043	0.034; 0.052	0.887	0.033	126.175	48	< 0.001

*CFI, comparative fit index; TLI, Tucker–Lewis Index; RMSEA, root mean square error of approximation; CI, confidence interval; pclose, probability of close fit; SRMR, standardized root mean square residual; df, degrees of freedom*.

**Figure 2:**
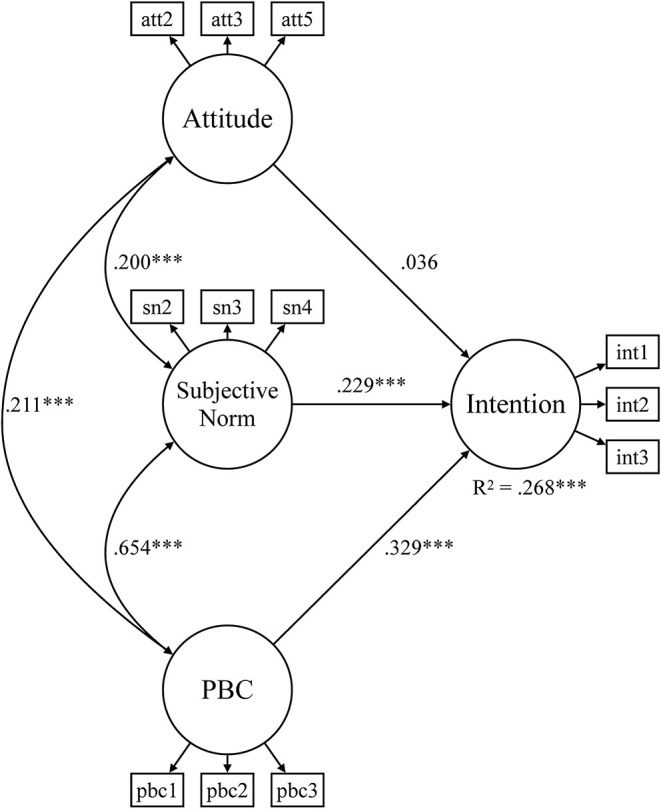
Structural equation model for environmentally friendly behavioral intentions of females. PBC, perceived behavioral control*;* ***p < 0.001.

**Figure 3:**
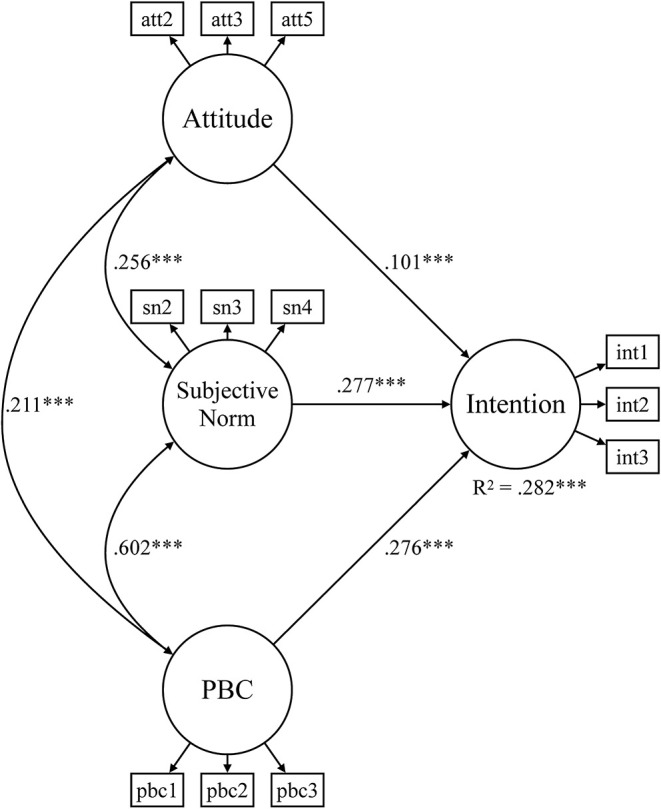
Structural equation model for environmentally friendly behavioral intentions of males. PBC, perceived behavioral control*;* ***p < 0.001.

A few gender- and sports-specific differences emerged in certain subsamples and parameters which are presented next. In the gender-specific subsamples (Figures 2, 3), 26.8 and 28.2% of the variation in behavioral intentions are explained by attitudes, subjective norms, and PBC, respectively. In the male subsample, all paths were significant, whereas the effect of attitudes on behavioral intentions was not significant in the women's sample.

**Figure 4:**
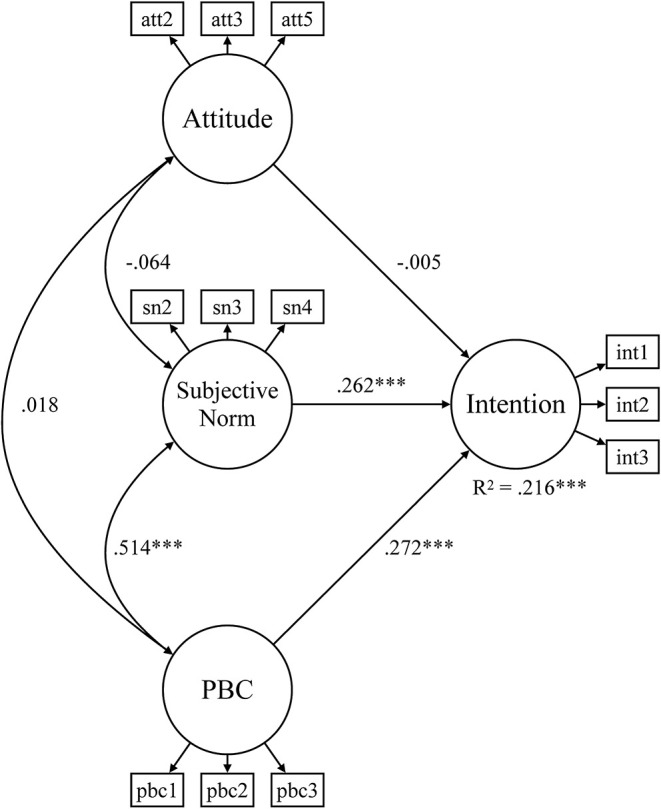
Structural equation model for environmentally friendly behavioral intentions of football club members. PBC, perceived behavioral control*;* ***p < 0.001.

Table 6 summarizes the results of the SEM for the sports-specific subsamples. It reveals that all correlation coefficients were statistically significant in subsamples for tennis and ice hockey. Accordingly, with 38.6%, the share of explained variance is higher in these sports than in the other three sports where it is between 20 and 25%. Insignificant effects are observed in handball, basketball, and football. In the handball subsample, all correlations are positive and significant with the exception of the effect of PBC on behavioral intentions. In the SEM for handball, all relationships are statistically significant and positive except for the effects of attitudes and subjective norms on behavioral intentions. As an illustrative example, the SEM for football is displayed in Figure 4. In this model, attitudes did not correlate significantly with subjective norms and PBC. Additionally, the effect of attitudes behavioral intentions was insignificant.

**Table 6:** Standardized regression coefficients, correlation coefficients, and explained variance for the structural equation models by type of sport.

**Parameter**	**Tennis**	**Ice hockey**	**Basketball**	**Handball**	**Football**
Attitude → Intention	0.098^*^	0.135^*^	0.194^***^	0.024	−0.005
Subjective norm → Intention	0.147^*^	0.308^***^	0.369^***^	0.142	0.262^***^
PBC → Intention	0.469^***^	0.305^***^	0.072	0.335^***^	0.272^***^
Attitude ↔ Subjective norm	0.439^***^	0.349^***^	0.227^***^	0.357^***^	−0.064
Attitude ↔ PBC	0.324^***^	0.374^***^	0.271^***^	0.374^***^	0.018
Subjective norm ↔ PBC	0.668^***^	0.638^***^	0.531^***^	0.661^***^	0.514^***^
*R*^2^ (Intention)	0.386^***^	0.386^***^	0.247^***^	0.204^***^	0.216^***^

*PBC, perceived behavioral control*;

* *p < 0.05*;

*** *p < 0.001*.

## Discussion

The purpose of this study was to enhance the knowledge about drivers behind behavioral intentions in relation to environmentally sustainable behavior and the resulting potential of grassroots sports clubs to contribute to the environmental sustainability of the sports sector. While previous studies focused on recycling intentions of sports spectators (e.g., McCullough and Cunningham, 2011) and intentions of pro-environmental behavior at home (Casper et al., 2020), this study advances our understanding of environmentally friendly behavioral intentions beyond recycling by also including sustainable consumption, energy saving, and transportation. Compared to other studies investigating antecedents of behavioral intentions in a sports setting (e.g., McCullough and Cunningham, 2011; Casper et al., 2020), the present sample can be considered large. Similar to other studies within this research context, males and younger people with higher education are better represented in sports and sports clubs compared to the German population (Federal Statistical Office, 2019; Wicker, 2019).

The first research question asked to what extent the antecedents of environmentally friendly behavioral intentions, as suggested by the TPB, can be applied to sports club members. Generally speaking, the TPB suggests that behavioral intentions are predicted by attitudes toward the behavior, subjective norms, and PBC. Overall, the SEM of the full sample (Figure 1) shows that these three antecedents significantly and positively predict the behavioral intentions of sports club members to act environmentally friendly within the next 2 weeks. This finding suggests that the TPB can be applied to sports club members and the grassroots sports context, respectively. Among the three antecedents, subjective norms were the strongest predictor of behavioral intentions. This finding echoes previous applications of the TPB in environmental research (Greaves et al., 2013) and sports research (Cunningham and Kwon, 2003; Chuan et al., 2014).

The second research question investigated gender-specific and sports-specific differences in the antecedents–intention relationship. While the male subsample showed significant paths for all three antecedents, the effect of attitudes on behavioral intentions was not significant in the female subsample. Recall that attitudes are linked to positive or negative experiences of individuals with a certain behavior and describe the internal component of intentions (Fishbein and Ajzen, 1977). One potential explanation for this finding is that within women, the higher caring ethic for the natural environment in the context of increasing environmental problems, especially regarding climate change, may lead to more depressive feelings and lower attitudes (Sakellari and Skanavis, 2013). It is possible that women's greater concern for the natural environment (e.g., Zelezny et al., 2000; Schultz, 2001), as also outlined in the concept of ecofeminism (Eagly and Wood, 1991; Ling, 2014), in addition with the negative experiences from the impacts of climate change could have led to resignation and, therefore, to the slightly lower attitudes of women club members. It is also possible that the context of sports clubs and the male environment plays a role. In the five-team and racket sports of the present study, men represent a substantially higher share among club memberships than women. For example, in Germany, the share of male club memberships is 60.0% in tennis and 62.1% in handball, 74.3% in basketball, and as much as 84.3% in football and 89.9% in ice hockey (DOSB, 2020). Hence, the present women club members are likely influenced by a male club environment which might have shaped their attitudes toward environmentally friendly behavior. In an effort to adjust to this male club environment, it is possible that women club members have overreacted by adjusting their attitudes toward environmentally friendly behavior even more downward than the male level.

Subjective norms were found to be higher for women than for men in the latent mean comparison. This difference can be explained by social role theory, holding that women and men are assigned specific roles that they are expected to fulfill and, therefore, also influence their social surrounding (Eagly and Wood, 1991; Wood and Eagly, 2012). Therefore, women could face stronger expectations and norms to act environmentally friendly (Economou and Halkos, 2020). Hence, we echo recent environmental research that also drew on social role theory to explain gender differences (Xiao and McCright, 2015; Milfont and Sibley, 2016; Economou and Halkos, 2020). One important aspect of social role theory is shared beliefs about gender in a society which emerge to gender roles. The attributes associated with gender roles “tend to be viewed as desirable and admirable for each sex, thereby adding prescriptiveness to gender roles” (Wood and Eagly, 2012, p. 70). Thus, the theory assumes that there are shared gender beliefs and, especially, a gender dichotomy. The latter was the basis for our statistical analyses. From certain perspectives of ecofeminism and social construction gender theory, this dichotomy and related gender beliefs should be deconstructed or at least be differentiated (Sturgeon, 2016; Estévez-Saá and Lorenzo-Modia, 2018). However, the present study was unable to take this aspect into account as the gender measure was binary in nature.

Another possible theoretical explanation relates to emotional empathy and social contexts. For instance, Milfont and Sibley (2016), as well as Arnocky and Stroink (2010), have shown that women tend to have higher emotional empathy than men do. In addition, Arnocky and Stroink (2010) also documented that emotional empathy predicts altruistic environmental concerns. This means that women are more concerned about environmental problems because of the consequences for others (Dietz et al., 2002). This relationship indicates that women tend to have a stronger orientation to the social contexts in the case of environmental issues. Since the subjective norm is based on the perception of the social environment, both higher emotional empathy and stronger altruistic environmental concerns of women could lead to higher subjective norms among women to act environmentally friendly.

Men showed higher PBC to act environmentally friendly. Recall that PBC is a self-assessment of being able to perform a behavior and not the behavior itself. Specifically, PBC can be defined as people's perceptions of the degree to which they are capable of performing a certain behavior and can also be seen as self-efficacy (Fishbein and Ajzen, 2010). Therefore, social role theory is one possible explanation for the higher PBC by men, since men are characterized as competitive, dominant, and masterful, which is related to higher self-confidence and self-efficacy of men (Eagly and Wood, 1991; Meyers-Levy and Loken, 2015). For example, Milfont and Sibley (2016) revealed that men tend to have a higher social dominance orientation than women. Consequently, in the present study, the higher PBC of men might suggest that they would be more likely than women to assume that it is under their control to act environmentally friendly in the context of the sports club and that they can do so whenever they want, regardless of other factors such as the social context. Therefore, social dominance orientation, as a stereotypical men-trait (Wood and Eagly, 2012), could be an explanation for the higher PBC of men.

The gender-specific latent mean comparison also revealed that women had higher intentions to act environmentally friendly in the foreseen future within their sports club. Based on the concept of ecofeminism, previous literature in environmental research suggests gender differences in environmental intentions due to different conceptualizations of the world, with the resulting closeness to nature leading to stronger caring for the natural environment (Leach, 2007; Sakellari and Skanavis, 2013). Hence, women's higher behavioral intentions to act environmentally friendly support the tenets of ecofeminism (Meinzen-Dick et al., 2014; Estévez-Saá and Lorenzo-Modia, 2018). Other perspectives of ecofeminism and feminist political ecology can also be advanced as explanations as they go beyond the closeness to nature and the focus on sustainability. According to Meinzen-Dick et al. (2014), the right to resources, the means and opportunity to exploit resources, and the adaption of sustainable practices are also relevant categories of ecofeminism and feminist political ecology. From these viewpoints, it is possible that women might have fewer resources to use in the first place. However, women are less likely to waste resources, indicating gender differences in the adoption of environmentally friendly behavior from a different perspective.

The evident gender difference in intentions of environmentally friendly behavior can also be interpreted through the lenses of social role theory and pro-social behavior. Environmentally friendly behavior qualifies as pro-social behavior (Schmitt et al., 2018). The latter is characterized by altruism, generosity, and helping others, including the natural environment. Likewise, and following social role theory, women are more likely to show these traits and are therefore more likely to demonstrate pro-social behavior and helping behavior, respectively (Fyall and Gazley, 2015). While the latter study was about volunteering (as another type of pro-social behavior), the present research suggests that the provided theoretical explanations apply to environmentally friendly behavior as well.

The results also showed differences in the antecedents–intention relationships among the five sports. While all three antecedents predicted behavioral intentions in tennis and ice hockey, PBC was insignificant for basketball and attitudes for handball and football. In handball, only PBC significantly predicted the intentions to act environmentally friendly in the next 2 weeks. These findings can be explained by Ajzen (1991) who proposed that the strength of the effect of the respective antecedent is situational and context dependent. Evidently, handball and football clubs represent a social context where attitudes toward a specific behavior do not translate into corresponding behavioral intentions. Moreover, subjective norms do not drive behavioral intentions in handball clubs and PBC does not affect behavioral intentions in basketball clubs.

The findings yield implications for policymakers and club managers. First, they suggest that not all genders and sports can be treated equally. Regarding gender, there is a need to recognize that women's behavioral intentions can only be increased by increasing PBC and subjective norms, not attitudes. The evident sport-specific differences can be used to develop specific initiatives to increase environmentally friendly behavioral intentions within specific sports. The strong effect of subjective norms on intentions of environmentally friendly behavior can be especially interesting for club managers across sports (except handball). Since grassroots sports clubs are characterized by strong social cohesion of members, a general awareness of club members for the importance of environmental topics can contribute to increasing environmentally friendly intentions of all members. Since club members are part of each other's social network, spillover effects can occur. Furthermore, handball and football club managers should give their club members enough opportunities to perform environmentally friendly behavior. Such opportunities might yield to higher PBC which has a positive effect on intentions and, later on, also on actual behavior (Webb and Sheeran, 2006). Collectively, sports policymakers should acknowledge that grassroots sports clubs can be considered an important context for increasing the environmental sustainability of the sports sector and the corresponding sustainable development goal.

## Conclusion

This study examined community sports club members' antecedents of environmentally friendly behavioral intentions as suggested by the TPB. Recognizing that sports clubs represent the most important provider of organized sports in many European countries, including Germany, their potential contribution to the environmental sustainability of the sports sector is acknowledged. Hence, it is critical to understand members' intentions of environmentally friendly behavior. The results of this study can guide policymakers and club managers to develop environmental measures that help achieve the 13th sustainable development goal by the UN and take urgent action to combat climate change and its impact.

The present work contributes to the increasing body of sports ecology research as well as TPB studies. This study was among the first to apply the TPB to sports environmental research. Furthermore, the largely neglected context of grassroots sports clubs within sports ecology was examined. Regarding the applicability of the TPB, the study made several contributions. On the one hand, the three antecedents successfully predicted the behavioral intentions of sports club members to act environmentally friendly, concluding that the TPB can be fully applied to sports club members and to the grassroots sports context. On the other hand, the study showed that there are sports-specific differences in the antecedents–intention relationship which can be used to increase the potential of grassroots sports clubs to contribute to the environmental sustainability of the sports sector.

This research comes with some limitations that could lead to future research avenues. While gender-specific differences could be explained by existing theories and concepts from gender and feminist literature, sports literature lacks theories outlining differences between sports. Hence, specific theoretical explanations for the differences that were evident between some sports are not available, indicating that the underlying reasons should be explored in future (qualitative) studies. Second, the study only investigates environmentally friendly intentions, not actual behavior. This limitation comes with the nature of the survey, which was anonymous. Thus, it is not possible to collect further data about actual behaviors and link these data with the existing dataset to examine how behavioral intentions have affected actual behavior a few weeks later. Future research wishing to compromise respondents' anonymity should investigate the intention–behavior relationship of community sports club members. Third, the study only investigated the antecedents–intention relationship based on the TPB and its constructs. Other context-specific variables, such as awareness of environmental issues, should be investigated in future studies.

## Data Availability Statement

The raw data supporting the conclusions of this article will be made available by the authors, without undue reservation.

## Ethics Statement

Ethical review and approval was not required for the study on human participants in accordance with the local legislation and institutional requirements. Written informed consent for participation was not required for this study in accordance with the national legislation and the institutional requirements.

## Author Contributions

MB drafted parts of the methods, conducted the data analysis, designed the graphical representation of the findings, and wrote parts of the results. TT drafted the theoretical framework and literature review, as well as parts of the methods, the discussion, and the conclusion. PW oversaw the data collection process and conducted parts of the data cleaning, drafted the introduction and parts of the results, discussion, conclusion, checked the overall manuscript for coherence, consistency, and format. All the authors contributed to the conception and design of the work, drafted it, revised it critically for important intellectual content, approved the final version of the manuscript, and agreed to be accountable for all aspects of the work in ensuring that questions related to the accuracy or integrity of any part of the work are appropriately investigated and resolved.

## Conflict of Interest

The authors declare that the research was conducted in the absence of any commercial or financial relationships that could be construed as a potential conflict of interest.
